# Characterizing Awareness of Schizophrenia Among Facebook Users by Leveraging Facebook Advertisement Estimates

**DOI:** 10.2196/jmir.6815

**Published:** 2017-05-08

**Authors:** Koustuv Saha, Ingmar Weber, Michael L Birnbaum, Munmun De Choudhury

**Affiliations:** ^1^ School of Interactive Computing Georgia Institute of Technology Atlanta, GA United States; ^2^ Qatar Computing Research Institute Hamad Bin Khalifa University Doha Qatar; ^3^ The Zucker Hillside Hospital Psychiatry Research Northwell Health Glean Olks, NY United States; ^4^ Hofstra Northwell School of Medicine Hempstead, NY United States; ^5^ The Feinstein Institute for Medical Research Manhasset, NY United States

**Keywords:** schizophrenia, psychotic disorders, online social networks, health awareness, mental health, public health, Facebook

## Abstract

**Background:**

Schizophrenia is a rare but devastating condition, affecting about 1% of the world’s population and resulting in about 2% of the US health care expenditure. Major impediments to appropriate and timely care include misconceptions, high levels of stigma, and lack of public awareness. Facebook offers novel opportunities to understand public awareness and information access related to schizophrenia, and thus can complement survey-based approaches to assessing awareness that are limited in scale, robustness, and temporal and demographic granularity.

**Objective:**

The aims of this study were to (1) construct an index that measured the awareness of different demographic groups around schizophrenia-related information on Facebook; (2) study how this index differed across demographic groups and how it correlated with complementary Web-based (Google Trends) and non–Web-based variables about population well-being (mental health indicators and infrastructure), and (3) examine the relationship of Facebook derived schizophrenia index with other types of online activity as well as offline health and mental health outcomes and indicators.

**Methods:**

Data from Facebook’s advertising platform was programmatically collected to compute the proportion of users in a target demographic group with an interest related to schizophrenia. On consultation with a clinical expert, several topics were combined to obtain a single index measuring schizophrenia awareness. This index was then analyzed for differences across US states, gender, age, ethnic affinity, and education level. A statistical approach was developed to model a group’s awareness index based on the group’s characteristics.

**Results:**

Overall, 1.03% of Facebook users in the United States have a schizophrenia-related interest. The schizophrenia awareness index (SAI) is higher for females than for males (1.06 vs 0.97, *P*<.001), and it is highest for the people who are aged 25-44 years (1.35 vs 1.03 for all ages, *P*<.001). The awareness index drops for higher education levels (0.68 for MA or PhD vs 1.92 for no high school degree, *P*<.001), and Hispanics have the highest level of interest (1.57 vs 1.03 for all ethnic affinities, *P*<.001). A regression model fit to predict a group’s interest level achieves an adjusted *R*^2^=0.55. We also observe a positive association between our SAI and mental health services (or institutions) per 100,000 residents in a US state (Pearson *r*=.238, *P*<.001), but a negative association with the state-level human development index (HDI) in United States (Pearson *r*=−.145, *P*<.001) and state-level volume of mental health issues in United States (Pearson *r*=−.145, *P*<.001).

**Conclusions:**

Facebook’s advertising platform can be used to construct a plausible index of population-scale schizophrenia awareness. However, only estimates of awareness can be obtained, and the index provides no information on the quality of the information users receive online.

## Introduction

### Background and Prior Work

Schizophrenia, although a relatively rare condition that occurs in approximately 1% of the world’s population, is associated with devastating individual, familial, and societal loss [1]. Psychotic symptoms, including hallucinations, delusions, and disorganized thinking and behavior, typically emerge during precious years of adolescent and young adult development interfering with the establishment of healthy social, educational, and vocational foundations [2]. The substantial burden imposed by schizophrenia has been linked to its early onset as well its incurable nature often associated with persistent psychotic symptoms. Schizophrenia is ranked among the top 25 leading causes of disability worldwide [1]. The World Health Organization has estimated that direct costs associated with schizophrenia in the United States range from 1.6% to 2.6% of total health care expenditures [3]. The economic burden of schizophrenia is found to be more than US $60 billion per year [4].

Despite effective treatment options, major impediments to receiving early and appropriate care include lack of public awareness, misconceptions and misunderstanding, and high levels of stigma associated with schizophrenia and other related behavioral health disorders [5,6]. Professional help is often only sought after very long delays or a crisis, contributing to negative outcomes and poorer response to available treatments [7]. Furthermore, once in treatment, stigma and poor public awareness and understanding of schizophrenia and other psychotic disorders leads to worse outcomes including lowered self-esteem, social withdrawal, poor self-care, and drug and alcohol misuse [8].

The importance of public awareness and understanding of schizophrenia has been repeatedly demonstrated in the scientific literature [9]. Mental health literacy is defined as the knowledge and beliefs about mental disorders which aid their recognition, management or prevention and includes the ability to recognize specific behavioral health symptoms and disorders as well as available treatment options [10]. Existing mechanisms to assess (and ultimately improve) population awareness of schizophrenia, however, are challenged by the difficulty in gathering reliable, near real-time, and fine-grained population data [11]. A number of mental health programs and campaigns tend to employ survey- and questionnaire-based approaches to assess awareness, but these approaches are often expensive, time consuming, and unable to reach a large nation-wide audience. Moreover, these approaches thus far have provided limited data on schizophrenia awareness in different demographic groups, such as sex, race, age, or geography. Finally, most awareness assessment surveys are administered with large temporal gaps. This practice poses challenges in the ability to act on awareness-related information for deploying appropriate intervention programs, improving mental health service facilities, or for conducting mental health awareness campaigns. In this paper, we aim to tackle this challenge by utilizing unobtrusively gathered social media (Facebook) data in the design and development of a population index of schizophrenia awareness.

There is now convincing evidence that social media activity can be used to reliably monitor health-related thoughts and behaviors [12-14]; this forms the basis of our work. From a public health perspective, social media data has been used to infer information ranging from the spread of the influenza virus across the United States to rates of seasonal allergies, human immunodeficiency virus (HIV) infection, smoking, depression, obesity, and a variety of other county-level health statistics [15-20]. Chunara et al [21] argued that crowdsourced data, gathered via new technologies including the Internet and mobile phones offer the opportunity to fill gaps in and augment current epidemiological models. Early work by Ginsberg et al [22] demonstrated this promise by employing Google’s search engine query data to monitor and track influenza trends in the United States.

There has been some work employing measurements of online activity, specifically Facebook “likes” as a mechanism for public health surveillance, and our work is motivated by these approaches. Gittelman et al [23] examined the predictive qualities of Facebook “likes” with regard to mortality, diseases, and lifestyle behaviors in 214 counties across the United States and 61 of 67 counties in Florida. Also employing Facebook “likes” as measures of topical interest, Chunara et al [24] found that activity-related interests across the United States and sedentary-related interests across New York City, inferred via Facebook’s advertisement estimate algorithm, were significantly associated with obesity prevalence. Other research has also been successful in accurately assessing personality traits, intelligence, happiness, substance use, sexual orientation, religious and political views from individuals’ “like” data on Facebook [25].

Prior work on the utility of social media activity data as a sensor of population-scale mental health awareness is limited. Korda and Itani [26] explored the role of social media as a platform for health promotion and behavior change, whereas Chou et al [27] explored this for health communication (also see [28]). Focusing on schizophrenia in particular, Birnbaum et al [29] studied how youth appropriated social media for seeking help around psychosis-related issues. Focusing on assessing the efficacy of awareness campaigns, Ayers et al [30] used the “Great American Smokeout” as a case study to observe cessation-related news reports and Twitter postings, and cessation-related help seeking via Google, Wikipedia, and government-sponsored quitlines. Other work conducted a randomized control trial to investigate the efficacy of an online depression awareness campaign that employed a Facebook-based recruitment strategy [31].

### Study Objectives

As demonstrated in prior work, Facebook “likes” (henceforth referred to as Likes) are a powerful means by which users can express their own thoughts and interests, including health concerns. We note that this data may also reveal important details about the type of information and health education an individual is receiving. For example, individuals who like a specific Facebook page, such as the public page titled “Schizophrenia Awareness” [32] will be provided with information from that source directed back to the user, in the case of this page the stated mission being “By spreading awareness among a large and popular Facebook community, we can help rid the world of the misunderstandings and misconceptions surrounding this condition.” Thus “Like” information can be particularly relevant for measuring schizophrenia awareness as it bears the potential to provide insight into the number of individuals who are expressing interest in or engaging with the topic of schizophrenia, as well as obtaining information related to schizophrenia through social media. Facebook additionally collects data on the interests of their users by passively monitoring any websites they visit, as long as those websites have Facebook Like or share functionality [33]. According to estimates, this kind of tracking happens on 75% of the 1000 most popular websites [34]. This information may further be useful to understand awareness around specific health topics such as schizophrenia. Together, in this paper, we employ these sources of information made available via Facebook to quantify population level awareness measures of schizophrenia among Facebook users in the United States.

Specifically, the three main objectives of our study are to

1. construct and examine an index that measures the awareness of different demographic groups around schizophrenia-related information on Facebook;
2. explore how this index differs across demographic groups and how it correlates with offline variables about population well-being; and
3. examine the relationship of Facebook derived schizophrenia index with other types of online activity as well as offline health and mental health outcomes and indicators.

Our specific source of data involves advertisement estimates of topics of interest made available by Facebook. Twitter, Facebook, and all other major social media platforms rely on targeted advertising for their revenue. To maximize the advertisement revenue, it is thus in the platform’s interest to learn as much as possible about their users. This way Facebook and others can provide advertisers with access to highly targeted custom audiences that meet certain criteria, including demographics as well as certain topical “interests.” According to Facebook’s Business page [29], “Interests may include things people share on their Timelines, apps they use, advertisements they click, Pages they like, and other activities on and off of Facebook and Instagram. Interests may also factor in demographics such as age, gender, and location.” Before an advertiser launches an advertising campaign, they are provided with an estimate of the audience size of the number of monthly active users on Facebook, so as to enable assessing the campaign’s cost. We note that this mechanism of gathering audience estimates essentially acts as an on-demand digital census, answering questions of the form “how many female Facebook users in the state of New York aged 25-44, have at least a bachelor’s degree and are interested in ‘schizophrenia?'” In this way, with data gathered through such queries, we can assess the level of interest or engagement of a particular demographic group in schizophrenia, psychosis, and related topics. We refer to this topical interest or engagement as “schizophrenia awareness” within the context of the objectives proposed in this paper.

## Methods

### Data Sources

#### Facebook Advertisement Estimates

The main source of data for our study (objectives 1-3) comes from Facebook’s Marketing application program interface (API) [35,36]. This API allows advertisers to limit their advertisement’s reach to a highly targeted, custom audience. As noted above, this custom audience can be tailored in terms of user demographics and topical interests [37]. Some of these variables, such as the age and gender, are self-declared by Facebook users, whereas in particular the topical interests are inferred automatically based on interaction both on and off Facebook [38]. As an example, the reader can check the list of inferred interests for their own Facebook profile online [39].

To obtain a list of “marker interests” that indicate a user’s interest in the schizophrenia topic, we made use of the (i) autocomplete and (ii) interest suggestion functionalities in Facebook’s Adverts Manager [40]. Through the autocomplete functionality, we obtained a full list of topics matching “schizophren*” and “psychos*.” This list was then pruned for relevance, removing topics such as “cosmic psychos.” We then selected the topic “psychosis” and used the suggested related interests to expand our list. Interests that matched too few users were dropped from consideration. Table 1 shows the final list of the five selected marker interests. To arrive at this final list, we used an iterative method of adding and pruning marker interests, closely engaging with our psychiatrist coauthor, who is an expert in schizophrenia.

**Table 1:** List of the schizophrenia-related “marker interests” used in our study.

Interest	Global user count	US user count
Psychosis	5.8M	1.5M
Schizophrenia awareness	4.0M	480K
Hallucination	1.7M	120K
Schizoaffective disorder	57K	9.3K
Paranoid schizophrenia	46K	7.5K

With the Marketing API, we obtained various audience estimates for a range of combinations of demographic variables in combination with the marker interests. Concretely, we “or”-ed the five marker interests as their distinction is not clear cut, especially for typical Facebook users. Note that, to protect against targeting individuals, Facebook’s Marketing API never returns values below 20, making 20 indistinguishable from 0. For our ensuing data analysis, tuples with an advertisement audience estimate of 20 were dropped from consideration.

Concerning demographic variables, we obtained separate audience estimates for (i) each of the 50 US states, (ii) both genders, (iii) five different age groups (13-17, 18-24, 25-44, 45-64, 65+ years), (iv) four different education levels (no high school degree; high school degree but no bachelor’s degree; bachelor’s degree but no master’s or PhD degree; master’s or PhD degree), and (v) four different ethnic affinities (Hispanic, African-American, Asian-American, none of the previous). For each of these dimensions, we also added a value of “all” which would define an audience across all possible values for that dimension. Note that Facebook carefully does not refer to “race” and that “ethnic affinity” is automatically inferred. So, a non-Hispanic user with a strong affinity toward Hispanic content and culture online might get labeled as Hispanic. Furthermore, no “White” ethnic affinity is offered. As of November 2016, Facebook has disabled the use of ethnic affinity for advertising related to housing, jobs, or credit, which have particular status concerning the protection against racial discrimination [41].

#### Google Trends

To get a comparative understanding with other complementary forms of online activity (objective 3), we obtained the US state-wise distribution of Google Trends [42] for the keywords present in our set of marker interests. Google Trends is a public Web facility of Google, based on their search engine, that shows how often a particular search-term (eg, “schizophrenia awareness”) is entered relative to the total search-volume (in a US state).

#### Offline Population Data

In addition to the above online data, we obtained different types of offline data, as a way to examine the relationship and validity of schizophrenia awareness obtained from Facebook with related health and mental health outcomes and indicators (objective 3). First, we obtained the number of mental health institutions in each US state from the substance abuse and mental health services administration (SAMHSA) database [43]. For the purposes of our ensuing analysis, we normalized these numbers per state by dividing them by the corresponding state’s population estimates (obtained from the United States Census Bureau [44]). Next, we intended to examine if factors like health, economy, and overall development of a state are associated with schizophrenia awareness. Therefore, we used the American human development index (HDI) of each US state, published by Measure of America, which is an initiative of the Social Science Research Council [45]. HDI is a composite index of life expectancy, education, and per capita income indicators. The American HDI is a modified version of the global HDI, using different indicators to better reflect the US context and to maximize the use of available data [46,47]. Intuitively, the HDI quantitatively summarizes the three dimensions of (i) health and well-being, (ii) knowledge, and (iii) standard of living. Third, we obtained the state-wise number of mental health issues among adults from The Henry J. Kaiser Family Foundation database [48].

### Assessing Validity of the Acquired Facebook Data

To what extent is the population-level data given by Facebook’s advertisement estimates representative of the US population? We note that it is important to assess the validity of the Facebook data we collected above before employing it in building a population index of schizophrenia awareness. For the purpose, we employed the same Marketing API to obtain the state-wise penetration of Facebook in United States. We obtained the number of users from the API for each state and computed their percentage on the corresponding population given by the US Census Bureau [44]. Based on this calculation, the national penetration of Facebook in United States was 63.16% and ranged from 56.35% (New Mexico) to 67.85% (Alaska), with a mean of 62.31% and standard deviation of 2.53%. This number is close to the Pew reported statistics of Facebook use in the US adult population (68%) [49].

Next, recall that one of our research objectives is to examine how awareness of schizophrenia varies across different demographic groups. However, one could anticipate that there are confounding factors between different demographic groups that may mediate this relationship; for example, young adults are perhaps more likely to be active Facebook users, leading to greater engagement manifested through Likes. To examine if these demographic group differences could be potential confounds, we again employed the Marketing API to evaluate the extent of activity of each demographic group on Facebook. Specifically, we obtained the count of audiences interested in either of entertainment, technology, music, and reading as marker interests; these topics were chosen because of the broad and popular scope. We then defined the percentage of active population as the ratio of the number of Facebook users with demographic attribute *d*, with at least 1 of 4 generic interests (entertainment, technology, music, and reading), to the number of Facebook users with demographic attribute *d.* We found that for each of the demographic attributes: state, ethnic affinity, education, and age, the standard deviation of the percentages of active population lied within 1.5-8%, indicating that there is little difference in activity across groups, or in other words, Facebook activity is likely not a confounding factor in our study.

### Index Construction

Our first research objective is to build an index which, for a given demographic group, measures the relative interest of that group in the topic of schizophrenia. Concretely, our SAI computes the proportion of the target demographic group, which has at least one of the marker interests:

SAI=(*n*_s_[*d*]/ *n* [*d*])×100

where *n*_s_(*d*) is the number of Facebook users with demographic attributes *d* and with at least one of the five schizophrenia marker interests, whereas *n* (*d*) is the total number of Facebook users with the same demographic attributes *d*. As an example, of the 19,000,000 women aged 18-24 years in the United States who use Facebook (*n* [*d*]), 230,000 have an interest in one of our schizophrenia interests (*n*_s_[*d*]). So the SAI for women, in the 18-24 age group is 1.21. Similarly, for any given subgroup defined in terms of gender, age, ethnic affinity, and education level, their count of users with a schizophrenia-related interest is divided by the number of Facebook users for the same subgroup.

### Statistical Models

First, to understand the interplay between different demographic variables and the SAI (objective 2), we computed and examined the trend of the index across changes in a single feature, such as across education levels or across ethnic affinities. Concretely, we used kruskal.test(...) function in R for Kruskal-Wallis rank sum test to compute *P* values and examine the statistical significance of data across demographic groups.

Next, to better understand the combined effects of several features (demographic attributes), we fitted linear regression models to predict the SAI for a given group. We experimented with two types of models, one including a binary indicator variable for each US state, and one where we replaced each state with their corresponding HDI, number of mental health institutions per 100,000 populations (MHP) and the percentage prevalence of mental health issues (MHI). The other independent variables for each of these models were the categorical features of age group, gender, ethnic affinity, and education. To ensure the stability of the model and the absence of statistical multicollinearity in the dataset, we computed the variance inflation factor (VIF). All computations were performed in R, using stats, reshape2, car, maps, maptools, and sp packages. We used coefficient of determination (*R*^2^) to understand the goodness-of-fit of the linear regression model, and *P* statistic to understand the significance of the variables.

Finally, toward our third research objective, we employed the use of correlation tests (Pearson product-moment correlation coefficient), which revealed the association between state-wise SAI and state-wise measures of searches given by Google Trends for all of the marker interests, the state-wise numbers of mental health institutions, state-wise HDI, and state-wise MHI.

## Results

### Measurements Enabled by the Schizophrenia Awareness Index

Toward our first research objective, we computed the SAI separately for each gender (male, female), for each ethnic affinity (African-American, Asian-American, Hispanic, none of the above), for each age group, in years (13-17, 18-24, 25-44, 45-64, 65+ years), and for each education level (no high school degree; high school degree but no bachelor’s degree; bachelor’s degree but no master’s or PhD degree; master’s or PhD degree).

**Figure 1:**
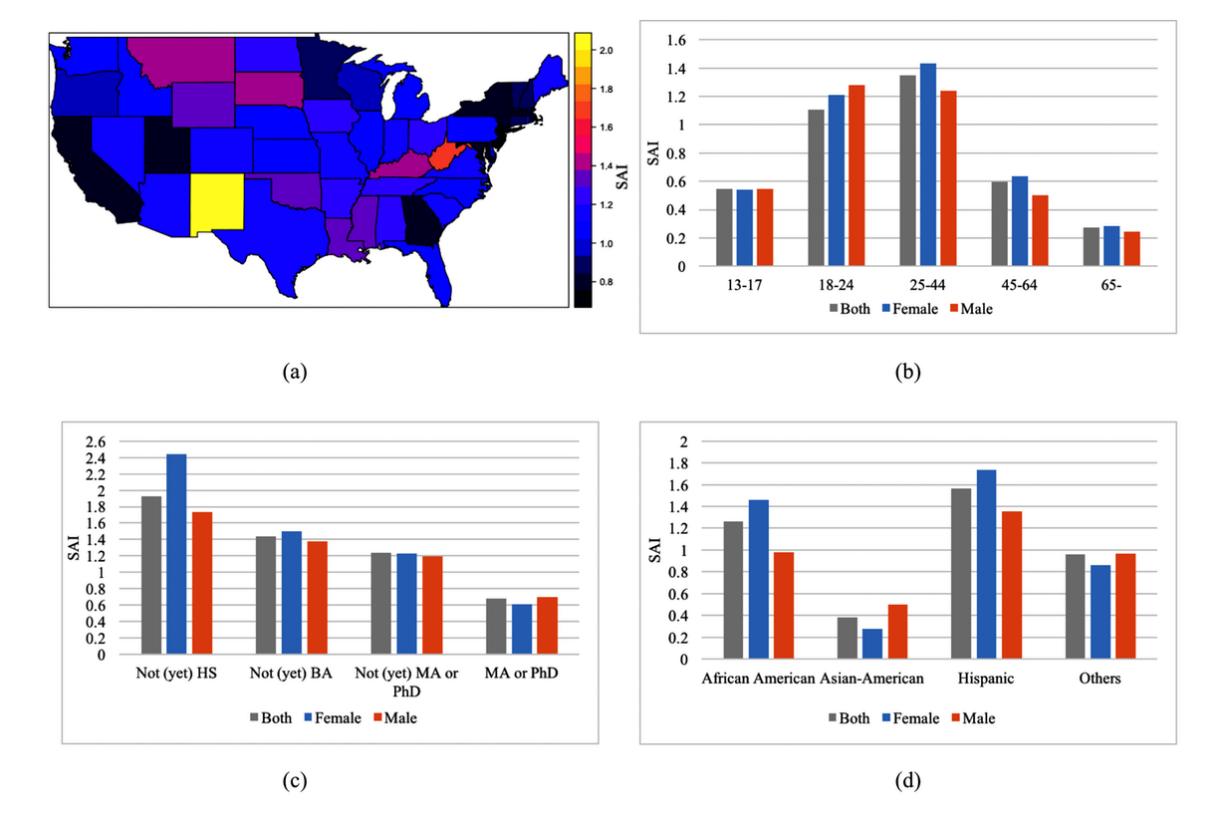
(a) Choropleth map showing the levels of schizophrenia awareness index (SAI) in different states of the mainland US; (b) distribution of SAI over age and gender; (c) distribution of SAI over education level and gender; and (d) distribution of SAI over ethnic affinity and gender.

We found the mean SAI across states to be 1.11, with a standard deviation of 0.24 (Figure 1 a). Among the states, New Mexico (2.00) and West Virginia (1.73) have the highest awareness index, whereas Maryland (0.76) and New Jersey (0.75) have the lowest awareness index. Concerning gender, we observed that females (1.06) have greater SAI than males (0.97, *P*<.001). The awareness index across age groups ranges from 1.35 (age 25-44 years) to 0.27 (age 65+ years), with a *P*<.001 (Figure 1 b). The index decreases with an increase in education level, from its highest of 1.92 for Facebook users with no high school degree, to its lowest of 0.68 for Facebook users having a master’s or PhD degree (*P*<.001) (Figure 1 c). Among ethnic affinities, the awareness index ranges from 1.57 (Hispanics) to 0.38 (Asian-Americans), with *P*<.001 (Figure 1 d). Each of these *P* values was computed using Kruskal-Wallis rank sum test.

### Analysis of Schizophrenia Awareness Index Over Demographics

In the previous section, we examined the interplay between our SAI and individual demographic variables. As a next step and per objective 2, we examine how particular *combinations* of demographic variables relate to our index. For this, we start by looking at all combinations of gender, state, age group, ethnic affinity, and education level for which there were at least 10,000 Facebook users. Table 2 lists the top 10 combinations in terms of their SAI.

We observe that the groups with highest SAI in Table 2 are dominated by demographic combinations of individual groups with a positive association with the SAI. For example, all the top 10 combinations are for women with Hispanic ethnic affinity and from one of the two age groups, 18-24 and 25-44 years, which also individually have the highest SAI scores. This indicates that the effect of the SAI is potentially “additive” allowing more in-depth analysis via a linear regression model.

Next, in order to examine the variability in SAI across demographic groups, we fit a linear regression model (described in the section “Methods”), with the SAI of a demographic group as a dependent variable, and using gender, age group, ethnic affinity, and education level as independent variables. Results of this model are given in Table 3. Especially for states with small populations, such as Wyoming or Vermont, using counts for, say, Hispanic women on Facebook aged 65+ years and holding a MA or PhD degree resulted in very low advertisement audience estimates. For the task of predicting the SAI for the remaining tuples, we obtained an adjusted *R*^2^=0.55 (*P*<.001), indicating a significant relationship between the independent and dependent variables. Table 3 lists a few of the significant variables (based on their *P* values). To ensure the absence of multicollinearity between variables, we computed the generalized variance inflation factors (GVIFs). The GVIF for each of the variables, ranged from 1.01 to 1.30 and the GVIF^(1/[2df]) (where df is the degree of freedom, or number of coefficients in the subset), ranged from 1.01 to 1.04. As these values are very close to 1.0, this indicates that there was no large multicollinearity in the linear regression model.

**Table 2:** Top 10 groups with highest schizophrenia awareness index (SAI) (at least 10,000 users).

Gender	State	Age (years)	Ethnic affinity	Education	SAI
Female	Ohio	18-24	Hispanic	Not (yet) BA	6.42
Female	Ohio	25-44	Hispanic	Not (yet) BA	6.36
Female	Colorado	18-24	Hispanic	Not (yet) HS	6.27
Female	Ohio	18-24	Hispanic	Not (yet) MA or PhD	5.91
Female	Kentucky	25-44	Hispanic	Not (yet) MA or PhD	5.88
Female	New Mexico	25-44	Hispanic	Not (yet) BA	5.52
Female	New Mexico	25-44	Hispanic	Not (yet) MA or PhD	5.47
Female	Ohio	25-44	Hispanic	Not (yet) MA or PhD	5.43
Female	Missouri	18-24	Hispanic	Not (yet) MA or PhD	5.38
Female	Michigan	18-24	Hispanic	Not (yet) BA	5.25

**Table 3:** Significant variables of Linear Regression Model 1 (dependent variable: schizophrenia awareness index [SAI]; independent variables: gender, state, age, education, and ethnic affinity).

Variable		Estimate (beta)		Std error		*t* statistic		*P* (>| *t* |)
(Intercept)		.00930		0.00170		5.5		<.001
**Gender**								
	Male		−.00448		0.00040		−11.1	<.001
**State**								
	California		−.00715		0.00173		−4.1	<.001
	Connecticut		−.00696		0.00203		−3.4	<.001
	Florida		−.00680		0.00179		−3.8	<.001
	Maine		.02120		0.00234		9.1	<.001
	Maryland		−.00687		0.00191		−3.6	<.001
	Montana		.02590		0.00225		11.5	<.001
	New Mexico		.00682		0.00200		3.4	<.001
	New York		−.00615		0.00177		−3.5	<.001
	South Dakota		.01970		0.00258		7.6	<.001
	Vermont		.01930		0.00287		6.7	<.001
	West Virginia		.02070		0.00213		9.7	<.001
	Wyoming		.01480		0.00265		5.6	<.001
**Age (years)**								
	18-24		.00914		0.00070		13.1	<.001
	25-44		.01110		0.00069		16.1	<.001
**Education**								
	Not (yet) BA		.00329		0.00073		4.5	<.001
	Not (yet) High School		.01020		0.00079		12.9	<.001
**Ethnic affinity**								
	Asian-American		−.00637		0.00095		−6.7	<.001
	Hispanic		.01330		0.00060		22.3	<.001
	Other		−.00549		0.00057		−9.6	<.001

In a second linear regression model (described within the section, “Methods”), we predicted the SAI using gender, age group, ethnic affinity, education level, as well as MHP, HDI, and MHI as independent variables. In contrast to the first model, we replaced the binary indicator variables for each state with their corresponding MHP, HDI, and MHI values. This model had an adjusted *R*^2^=0.56 (*P*<.001). The GVIF for each of the variables ranged from 1.01 to 1.70, and the GVIF^(1/[2×df]), ranged from 1.00 to 1.07, again indicating the absence of multicollinearity in the linear regression model. Table 4 lists the significant variables (based on their *P* value).

Though the adjusted *R*^2^ for the second model was higher than for the first model, we still wanted to examine the role played by a state’s mental health issues and infrastructure or overall development. So, we fit a third linear regression model using only gender, age group, ethnic affinity, and education as independent variables, that is, not using either of state or MHP or HDI as predictors. We observed a reduced adjusted *R*^2^ of 0.42, compared with 0.56 for the previous model with MHP, HDI, and MHI included.

### Relationship of SAI With Online Activity and Offline Health Indicators

Toward our third and final research objective, we first, we performed correlation tests for MHP, HDI, and MHI with SAI, and Table 5 shows the results. On the basis of these results, we can confirm that each of MHP, HDI, and MHI has an association with SAI. In the case of MHP, presence of more mental health institutions is *positively* associated with SAI. However, an increase in the HDI is *negatively* associated with SAI. For MHI, we observe that prevalence of mental health issues in different states is negatively correlated with SAI.

**Table 4:** Significant variables of Linear Regression Model 2 (dependent variable: SAI [schizophrenia awareness index]; independent variables: gender, MHP, HDI, MHI, age, education, and ethnic affinity).

Variable		Estimate (beta)		Std error		*t* statistic		*P* (>| *t* |)	
(Intercept)		.02181		0.00535		4.1		<.001	
**Gender**									
	Male		−.00183		0.00080		−2.3		<.001
**MHP^a^**		.00199		0.00016		12.3		<.001	
**HDI^b^**		−.00364		0.00055		−6.6		<.001	
**MHI^c^**		−.02740		0.01266		−2.2		<.001	
**Age (years)**									
	18-24		.01463		0.00123		11.9		<.001
	25-44		.01642		0.00124		13.3		<.001
**Education**									
	Not (yet) BA		.00717		0.00119		6.0		<.001
	Not (yet) HS		.01448		0.00126		11.5		<.001
	Not (yet) MA or PhD		.00507		0.00119		4.3		<.001
**Ethnic affinity**									
	Asian-American		−.01052		0.00148		−7.1		<.001
	Hispanic		.01887		0.00108		17.5		<.001
	Other		−.00414		0.00096		−4.3		<.001

^a^MHP: number of mental health institutions per 100,000 population.

^b^HDI: human development index.

^c^MHI: percentage of reported mental health issues in each US state.

**Table 5:** Pearson product-moment correlation of schizophrenia awareness index (SAI), with number of mental health institutions per 100,000 population (MHP), human development index (HDI), and volume of mental health issues (MHI) in each US state.

Metric	MHP vs SAI	HDI vs SAI	MHI vs SAI
Pearson *r*	.238	−.145	−.279
95% CI	0.206-0.270	−0.179 to −0.112	−0.516 to 0.001
*t* statistic	14.07	−8.44	−1.99
*P* value	<.001	<.001	.05

**Table 6:** Pearson product-moment correlation of schizophrenia awareness index (SAI), with Google Trends results of search of schizophrenia-related marker interests for each US states.

Google Trend search	Schizophrenia	Psychosis	Schizoaffective disorder	Hallucination	Paranoid schizophrenia
Pearson *r*	.31	.17	.28	.14	.46
Degree of freedom	48	48	40	43	36
95% CI	0.04-0.55	−0.11 to 0.43	−0.02 to 0.54	−0.16 to 0.41	0.17-0.68
*t* statistic	2.29	1.17	1.87	0.91	3.13
*P* value	.03	.25	.069	.34	.003

Next, for comparing state-wise SAI and the state-wise statistics of the marker interests given by Google Trends, we computed the correlation between the percentile Google Search of our marker interests for each state with the corresponding SAI. Table 6 lists the results. These results show that there is some harmony between the two online activity detection platforms (especially for marker interests Schizophrenia and Paranoid Schizophrenia).

## Discussion

### Principal Findings

Our work shows that data obtained from pervasive social technologies like Facebook may be employed to track public health awareness around schizophrenia-related concerns. This form of tracking needs little active participation and intrusion, and can be performed at a fine temporal, spatial, and demographic granularity at little resource cost. This makes our proposed index a promising mechanism for monitoring the impact of mental health awareness campaigns in near real-time, instead of through retrospective approaches such as surveys that are prone to hindsight bias of responders as well as experimenter demand effects. Prior work [34] reveals that the advertisement estimates are updated weekly, enabling this type of near real-time monitoring; we include a link [50] as a prototype application for the purpose. Additionally, our index could be used to craft more tailored awareness programs, targeting specific demographic groups with low levels of schizophrenia awareness.

Our results raise some interesting discussion points related to population-scale awareness of schizophrenia. First, due to the unavailability of gold standard data on schizophrenia awareness in the general population, we were not able to validate our proposed index with direct correlation metrics. However, our statistical examinations of SAI with three population-scale variables, availability of mental health services (or presence of mental health institutions), the HDI, and the proportion of mental health issues reported, show that the levels of schizophrenia awareness given by our approach bears relationship to offline variables of population well-being in different states of the United States.

On the above note, a priori, it is not clear if there should be a positive or a negative association between the presence of mental health institutions and our proposed SAI. A negative link could indicate that limited access to mental health institutions forces residents to go online to obtain information, whereas a positive link could indicate that more access helps to promote visibility and awareness. In our data, we find a robust positive link between the SAI and the MHP (see Tables 4 and 5). Similarly, both a positive association between the HDI and our SAI, as a result of better education and general interest in health topics, or a negative association, due to the availability of traditional information sources, are plausible hypotheses. In addition, association between SAI and the MHI could be either due to more number of mental issues leading to higher awareness, or due to higher awareness leading to decreased number of issues in mental health. Our results indicate a negative link between SAI and the MHI (see Tables 4 and 5). Further research is needed to derive causal explanations behind the observed directionality of these correlational relationships between SAI and population well-being variables.

### Comparison With Prior Work

Although there is a lack of prior work employing social media data to assess schizophrenia awareness, we situate our findings in the light of clinical, psychiatric, and public health research on mental health and schizophrenia awareness. A notable finding given by analyses enabled by our index is the heightened awareness among females compared with males. It is known from prior work that gender and age influence intentions to seek professional psychological help [51]. Females exhibit more favorable intentions to seek help from mental health professionals than males, likely due to their positive attitudes concerning psychological openness [52, 53]. This may explain our observed higher SAI in females over males.

Our results further indicate age-related differences in schizophrenia awareness among females and males. Specifically, SAI for females is highest in the age group 25-44 years, whereas for males, it is highest in the 18-24 age group. It is known that typically schizophrenia develops in late adolescence and early adulthood, and its clinical onset is observed to be later for females compared with males [54]. Consequently, it is possible that the awareness of the condition will correlate to the timeframe individuals are typically known to be affected by the illness.

Furthermore, we found Hispanics to be the ethnic affinity with the highest SAI (1.57 vs 1.03 for all ethnic affinities, *P*<.001). However, according to a study on the demographics of mental health service users [55], Hispanics are the ethnicity with the lowest use of mental health services. The same study also found that they are the group that is most likely to mention “prejudice and discrimination” as a reason for not using mental health services. This discrepancy may indicate that this ethnic group is appropriating online sources (namely, Facebook) more extensively to gain information regarding schizophrenia, possibly because they provide a prejudice, bias, and stigma-free mechanism to gain mental health literacy and information.

Of note, we find that the overall schizophrenia awareness given by our index is 1.03, which is close to the prevalence of 1.1% given by the National Institute of Mental Health (NIMH) [56, 57]. One possible explanation behind this observed alignment could be that the set of people who express schizophrenia interest on Facebook (eg, by “liking” different pages on the topic), also tend to be the ones who are themselves suffering or have suffered from schizophrenia or related psychotic disorders. However, further research through validated self-reported or clinical assessment of mental health is required to reveal to what extent schizophrenia awareness derived from online data sources actually maps to schizophrenia prevalence.

### Limitations

Our analysis relies on data provided by Facebook. Though this data is large-scale in its size and its granularity, it lacks transparency concerning how exactly Facebook collects this data. In particular, it is only partly clear how a user’s “interests” are inferred. This reliance on a “black box” is not desirable and related limitations have been discussed in the context of the now obsolete Google Flu service [58]. One consequence of hidden algorithmic details is that we observed certain fluctuations and inconsistencies. As an example, Figure 1 for the age group of 18-24 years shows a SAI value of 1.11, whereas the value for each gender separately in the same age group is higher (1.21 for female and 1.28 for male). The reason for this is that the advertisement audience estimate for the specification regardless of gender gave a lower count (410,000 out of 37,000,000) than the two counts separately (230,000 out of 19,00,0000 for female and 230,000 out of 18,000,000 for male). Though these inconsistencies were generally small, they show that the numbers one obtains might not be as reliable as one might assume.

But, even if we knew how exactly Facebook infers a user’s interests and how they perform their audience estimates, there would still be the question of what this *means*. Does the interest in a disease indicate that the user is suffering from it? Does it indicate that someone in the user’s social circle is affected by it? Or, does it just indicate general awareness of a topic? Future work can help to shed light on this question by looking more closely at the link between interest levels and prevalence rates. Relatedly, although we found statistically significant relationships between SAI and offline variables like state-wise number of MHP or HDI, further research is needed to understand the implications of these relationships.

Though this work uses Facebook data, other platforms such as Twitter also support targeted advertising and provide an audience estimate. Over time we expect the reach and the accuracy of such “digital census” tools to improve, supporting a wide range of public health monitoring capabilities. But on top of the passive use for data monitoring, targeted advertising or respondent driven sampling techniques can also be used for recruiting study or survey participants [59, 60].

Despite the growing use of social media and its potential as a source of population level data, it is important to note that large parts of the population still do not use it. Relying on data from social media to inform policy decisions can hence lead to a biased view on the world, caused by the digital divide [61]. In fact, even if a particular group is on social media, data analytic algorithms might not perform adequately for members of this group. As an example, the detection of schizophrenia-related interests might perform worse for minority languages where less training data is available, a case of “algorithmic bias” [62]. Similarly, not all individuals report their demographic attributes on Facebook, or include sufficient signals through their Facebook activity that may lend toward advertisements estimates computed by Facebook or the awareness measures given by our approach. We acknowledge a self-selection bias in our data and therefore the assessments made.

Finally, the work described so far only considers the aspect of *monitoring* schizophrenia awareness, but not *improving* it. However, the platform we are using to obtain our data is ultimately designed for exactly that getting a particular message out to a highly targeted audience. Although running a campaign with the goal of attracting millions of advertisement clicks might be beyond the practical feasibility of typical public health campaign budgets, a smaller pilot campaign, in the form of a randomized control trial, may be used to assess the right kind of message to use on other media. Furthermore, the Facebook advertisement platform can be leveraged to reach out to a variety of users, through a survey so as to also validate and calibrate the levels of awareness of schizophrenia among different groups. These constitute promising directions for future research.

### Conclusions

In summary, though more work on validation remains, we believe that our work indicates that there is clear potential in using unobtrusively collected data from social media for monitoring awareness of stigmatized health conditions such as schizophrenia and potentially others as well.

## Abbreviations

API: application program interface
GVIF: generalized variance inflation factor
HDI: human development index
HIV: human immunodeficiency virus
MHI: percentage of reported mental health issues in each US state
MHP: number of mental health institutions per 100,000 population
NIMH: National Institute of Mental Health
SAI: schizophrenia awareness index
SAMHSA: substance abuse and mental health services administration
VIF: variance inflation factor
